# Methodological approach for fast high-resolution image selection: FAHRIS algorithm

**DOI:** 10.1016/j.mex.2024.103072

**Published:** 2024-11-28

**Authors:** Bjørnar Åsebø, Stefano Cavazzani, Chiara Bertolin, Carlo Bettanini, Giampaolo Fusato, Andrea Bertolo, Pietro Fiorentin

**Affiliations:** a Department of Mechanical and Industrial Engineering, Norwegian University of Science and Technology, Trondheim, Norway; b Department of Physics and Astronomy, University of Padua, Padua, Italy; c INAF, Osservatorio Astronomico di Padova, Padua, Italy; d Department of Industrial Engineering, University of Padua, Padua, Italy; e CISAS – Center for Studies and Activities for Space Giuseppe Colombo, University of Padua, Padua, Italy; f Regional Environmental Prevention and Protection Agency of Veneto, Padua, Italy

**Keywords:** Site testing, Light pollution, Atmospheric effects, Radiosondes, Unmanned aerial vehicles, Image analysis, Image classification, Image grouping, Image stitching, Fast Algorithm for High-Resolution Image Selection - FAHRIS

## Abstract

Recent research highlights advancements in collecting Artificial Light at Night (ALAN) data using radiosondes on stratospheric balloons, revealing a need for enhanced in-flight image stabilization. This paper proposes a twofold approach: Firstly, it introduces a design concept for a high-resolution image acquisition and stabilization system for aerial instruments (e.g., drones, balloons). Secondly, it presents a novel Fast Algorithm for High-Resolution Image Selection (FAHRIS) for rapid image selection, grouping and stitching of acquired imagery. FAHRIS’ effectiveness is validated using datasets from three flights over Italy: a stratospheric balloon flight reaching 34 kms over Florence, and drone flights using a DJI Mavic 2 up to 253 m over Trevisoand 330 m over Padua. Limitations and challenges encountered during the validation of FAHRIS, such as computational constraints affecting dataset processing, are addressed. Additionally, the results of the image stitching process highlight potential distortions and stretching issues, particularly evident in images with significant relative angles.

• Design proposition of stabilization system for aerial instruments.
• Development of a novel and fast image selection, grouping and stitching algorithm (FAHRIS).
• Validation of algorithm against data sets from three flights.

## Graphical abstract

**Figure:**
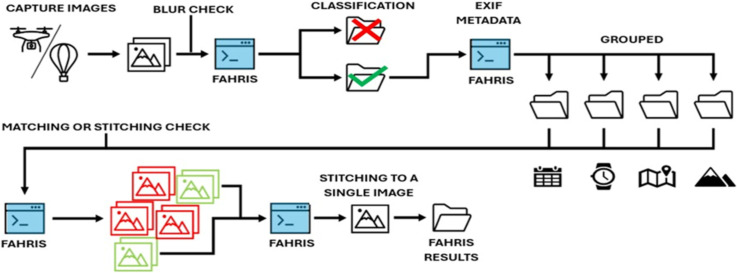

Specifications table

**Table:** 

Subject area:	Environmental Science
More specific subject area:	High resolution image study
Name of your method:	Fast Algorithm for High-Resolution Image Selection - FAHRIS
Name and reference of original method:	N/A
Resource availability:	Python code avaliable on Zenodo: https://doi.org/10.5281/zenodo.11109445

## Background

Recent advancements in the field of Artificial Light at Night (ALAN), as demonstrated by research by Cavazzani et al. [1], and Bettanini et al. [2,3], showcase the successful collection of ALAN data for the study of light pollution using a radiosonde attached to a stratospheric sounding balloon at 30 km altitude. Instrument calibrations used in these observation campaigns and for data acquisition on ALAN is an important area of research [[4], [5], [6]]. These studies on ALAN primarily aim to better understand and mitigate its impacts on astronomy, human health, and environmental well-being. From these experiments, data indicate the need for improvements of in-flight image stabilization to enhance data quality. In this contribution, concepts for lightweight, cost-effective, and improved in-flight image stabilization are presented in addition to a second objective focusing on the development of an automated Fast Algorithm for High-Resolution Image Selection (FAHRIS) analysis algorithm for acquired data. The intent of FAHRIS is to streamline the interpretation and categorization of images, including classification, grouping, and stitching. In this paper, the FAHRIS algorithm will be validated against data from both stratospheric balloon and UAV flights. An important aspect of FAHRIS compared to other image classification algorithms is its versatility and ability to operate with low-cost instrumentation and limited computing power. The code used can simplify the FAHRIS algorithm and stop the procedure after the first motion blur classification, or after clustering or matching, depending on the analysis needs. Other algorithms, in fact, are aimed at a single type of classification (e.g. motion blur classification [7,8], grouping [9,10] or stitching [11,12]) and use sophisticated tools and methodologies that require large computing capacity. The comparison with the individual steps positions FAHRIS as a fast, economical, complete and accessible procedure for a pre-analysis of big image quantities resulting from observation campaigns. High-performance, expensive algorithms and instruments have more specific and less versatile applications.

## Method details

### Concepts for image and camera stabilization system on equipped flying instruments

**Fig. 1:**
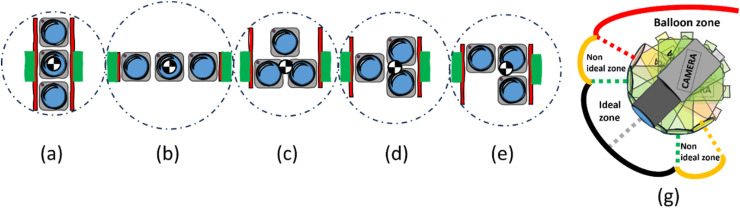
In (a) to (e): illustration of various camera array options. (g): Illustration of the different camera pitch zones.

In the following, five camera arrays ((a), (b), (c), (d), (e)) will be presented, illustrated in Fig. 1. Each setup's center of gravity is symbolized and aligns with the green hinges. Red lines represent brackets connecting the cameras and hinges, while circles indicate their rotational positioning around the center of gravity. The radiosonde uses a horizontal array (b) which allows for easier lens adjustments, a benefit also seen with the vertical array (a). The pyramid ((c) and (d)) and l-shaped (e) arrays offer a more compact design but may prove challenging in lens adjustments compared to the horizontal (b) and vertical (a) configurations. Both pyramid ((c) and (d)) and l-shaped (e) arrays can flip or rotate to adapt to space constraints, providing flexibility. As for drones, various configurations are utilized depending on the manufacturer, e.g. the DJI Mavic 3^1^ Pro utilizes an inverted version of (c), meanwhile the DJI Mavic 2^2^ only utilizes one camera.

In prior tests done by Bettanini et al. in 2022 with a camera array mounted on a radiosonde carried by a stratospheric balloon [2], the radiosonde's pitch was within ±10°, establishing the ideal zone for stabilization. In (g), Fig. 1, the ideal zone marked in grey is where the camera stays within for most of the flight. The non-ideal zones (> 10° from center) marked in yellow will therefore be the ideal location for stopping the camera from pitching. The balloon zone marked in red is the zone where the camera should not be able to pitch as it points directly to the balloon, rendering images unusable for further analysis. To prevent the cameras from entering unwanted zones, mechanical restrictions such as incorporating dampeners like rubber pads, foam, or springs can be implemented.

**Fig. 2:**
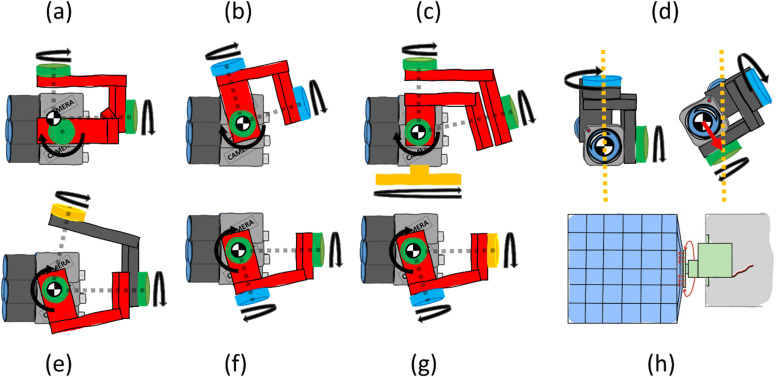
(a): Illustration of mechanical stabilization using a gimbal, utilizing electric motors, marked in green, actively controlling each axis’ rotation. (b): Illustration depicting a gravitational concept with free-rotating hinges, highlighted in blue and an electric motor, indicated in green, employed for active pitch control of the cameras. (c): Illustration showcasing the concept of incorporating free-moving hinges (highlighted in green) and a gyroscope (designated in orange). (d): Comparison between a stationary and a rolling camera is depicted in the illustration. (e) to (g): Illustration of different configurations of the camera stabilization system. (h): Illustration of the controllable wing concept. The servomotor is highlighted in green, the wing in blue, and the radiosonde's body in grey.

To maintain the camera's orientation during the radiosonde's movements, it is essential to enable movement along the lateral, longitudinal, and perpendicular axes. This requires integrating joints to adjust orientation using gravitational or mechanical forces. In the following, concepts utilizing gravity, gimbals, viscous dampeners, gyroscopes, and wings will be showcased. These concepts are illustrated above in Fig. 2.

In (a), a **gimbal configuration** with three electric motors (marked in green) actively controlling each axis’ rotation to ensure camera stabilization. This stabilization mechanism is commonly used for cameras on drones. Strategically positioned and centered on the respective rotation axes, these motors require minimal torque for balanced and responsive stabilization, enabling precise control over the camera's movement in multiple directions. The synchronized action of the electric motors within the gimbal offers a versatile and effective solution for camera stabilization. Various configurations of gimbals (green), viscous dampeners (blue), and free-moving hinges (orange) are depicted in (e), (f) and (g).

Utilizing **free-rotating** hinges it enables passive stabilization, utilizing gravity to return the system to its normal position during rotation. In (b), a system combines free-rotating hinges (highlighted in blue, facilitating passive stabilization along the roll and yaw directions (longitudinal and perpendicular axes)) with an electric motor controlling the camera's pitch (colored in green). In (d), the red arrow illustrates how the resultant moment corrects the camera's rolling motion toward the center of gravity, illustrated with a dotted orange line. Replacing free-rotating hinges with **viscous dampeners** enhances the system by smoothing movement and reducing the pendulum effect. Passive stabilization persists while the dampeners absorb and dissipate energy during rotation, ensuring a controlled return to the normal position. This adaptation optimizes stabilization, resulting in smoother motion responsive to gravitational forces.

Building on the same setup with free-moving hinges (highlighted in green), integrating a **gyroscope** (designated in orange) beneath the camera enhances stabilization (see Fig. 2(c)). The gyroscope uses angular momentum principles to counteract unintended movements, actively sensing and responding to deviations to keep the camera steady. This combined approach strengthens the system's resilience and reliability in diverse conditions. To mitigate yaw rotation, two **controllable wings** with servo motors can be integrated into the system as in Fig. 2(h). GPS heading data dynamically determines necessary adjustments, supported by accelerometer measurements to calculate precise wing adjustments. Leveraging this data, the system achieves effective and responsive control to enhance overall stability.

### Fast algorithm for high-resolution image selection – FAHRIS

The methodological approach for the fast elaboration of the high-resolution acquired images i.e. the novel Fast Algorithm for High-Resolution Image Selection - FAHRIS follows a step-by-step approach. The FAHRIS algorithm allows for quick classification, grouping and stitching of images acquired with cameras mounted on airborne vehicles such as radiosondes or UAVs (e.g. drones). In the subsequent sections, the structure and function of the algorithm is further detailed.

### Classification

To improve the analysis of the acquired images, poor-quality images should be disregarded. As an example, instability during the camera's time of exposure causes motion blur in the acquired image and thus very poor quality. Therefore, looking at the image's sharpness is one way of classifying stability and consequently of discriminating high- from poor-quality images. The sharpness can be calculated by analyzing highly frequent components in the images, using OpenCV,^3^ a Python pack that works on images and videos, giving various specters of functionalities to process, analyze, and manipulate data such as masking and blending, filtering, picture segmenting, and feature detection and matching.

**Fig. 3:**
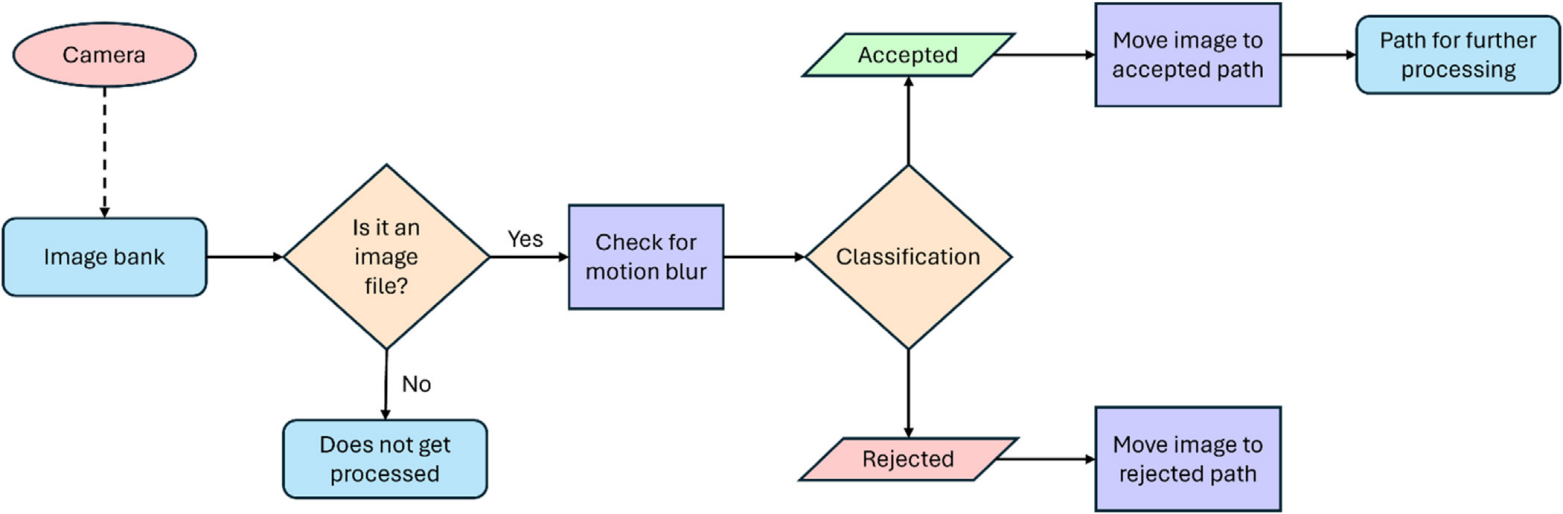
Structure of the FAHRIS classification algorithm.

The FAHRIS classification algorithm is designed to evaluate a repository of raw, unprocessed images. Its primary function is to analyze these images for the presence and severity of motion blur and classify them to either be accepted (high-quality acquired images) or rejected (poor-quality acquired images) based on the severity of the detected motion blur. The FAHRIS algorithm's systematic classification workflow is illustrated in Fig. 3.

To assess the presence of motion blur, a Laplacian operator is used for edge detection.^4^ This highlights areas of rapid changes in intensity on the image. This variation of intensity from the mean is denoted as variance and serves as a measure of the amount of the image's detail and sharpness.^5^ Using the variance, a threshold value is established to classify images based on their level of motion blur. Subsequently, FAHRIS compares the variance of each image to the determined threshold value to assign a classification. In FAHRIS, only one threshold value is established, thus it classifies images as “accepted” or “rejected”. Meanwhile, it is possible to establish several threshold values, e.g. value ranges that classifies for manual review. Images with variances lower than the threshold are considered to have reduced sharpness and are classified as “rejected”. Depending on the classification, the program moves each file to its respective directory. The FAHRIS classification algorithm is executed for each image file in the raw directory, systematically classifying images based on their motion blur characteristics. Furthermore, the FAHRIS classification algorithm has been designed to focus on motion blur and does not account for objects obstructing the focus area such as clouds.

### Grouping

Grouping data is a strategy for efficient organization and fast analysis. Grouping procedures can be used to locate data in the data set from a given value, achieving local aggregated data enabling contextual analysis and aiding in targeted processing. There are various values which can potentially be used as the basis for grouping, such timestamps, altitudes, geospatial, temperature and humidity data, as further detailed below.

Timestamps-based grouping allows for grouping by intervals (e.g. every 15 min) or time windows (e.g., between 01:15 - 01:35 and 01:40 - 02:00). Altitude-based grouping organizes images by elevation. This allows for grouping of given altitude ranges or grouping based on the ascend rate, resulting in groups of images containing relevant and comparable data. Geospatial data sets benefit from grouping based on longitude and latitude. This allows for grouping of data points according to their geographic coordinates, or according to their positional change from each other. Temperature-based grouping enables analysis within specific temperature ranges. This could unveil temperature-dependent patterns and trends, proving valuable in climate studies and environmental monitoring. Humidity-based grouping allows targeted analyses, exploring variations and patterns specific to different humidity ranges. This could prove beneficial in understanding atmospheric conditions and their implications on diverse applications.

The data used by FAHRIS algorithm for the grouping relies on the EXIF metadata extracted from each image. This metadata offers detailed insights, including the geographic location and altitude where the image was captured, the date and time of capture, along with various camera settings employed at the moment of capture.

In this paper, the grouping in FAHRIS is based on the **date, time, altitude**, and **geospatial data**. For the grouping by **date and time**, the data was grouped by daily intervals, with further subdivision into 15-minute segments within each day. For the grouping based on **geographical coordinates**, a systematic grid-based approach was adopted. This spatial grid consists of sectors measuring 10 by 10 s. It extends across the entire range of recorded longitudinal and latitudinal data points. Each sector corresponds to a defined range beginning with the minimum coordinate value and ending with the maximum. The sectors are defined in increments of 10 s along the longitude. For each longitudinal increment, a corresponding latitudinal sector is defined in 10 s. The sequence of these increments follows the pattern exemplified in Table 1 below where n=0,10,20…n·10.

**Table 1:** Structured representation of a spatial grid system for geographic coordinates [degrees (°), minutes (‘), seconds (“)], divided into 10 by 10 second sectors. Each cell corresponds to a specific sector within these defined ranges, creating a systematic grid for mapping and analysis.

	Longitude [°, ‘, “]					
Latitude [°, ‘, “]	$lon_{0\to 9}$	$lat_{0\to 9}$	$lon_{10\to 19}$	$lat_{0\to 9}$	$lon_{n\to n+9}$	$lat_{0\to 9}$
	$lon_{0\to 9}$	$lat_{10\to 19}$	$lon_{10\to 19}$	$lat_{10\to 19}$	$lon_{n\to n+9}$	$lat_{10\to 19}$
	$lon_{0\to 9}$	$lat_{n\to n+9}$	$lon_{10\to 19}$	$lat_{n\to n+9}$	$lon_{n\to n+9}$	$lat_{n\to n+9}$

At the equator, a sector equals an area of approximately $309x309\;m^{2}$. Due to convergence of meridians, sectors closer to the poles will have reduced longitudinal dimensions resulting in a rectangular shape. At specific sampling locations each sector will nonetheless maintain a nearly identical configuration, providing a consistent grid.

The images have additionally been systematically grouped by FAHRIS based on their **altitudinal positions**, segmented into intervals of 50 m ($h_{0\to 49},h_{50\to 99}\ldots \;h_{n\to n+49\;}$where n=0,50,100…n·50). Although no further subgrouping within these has been implemented, it is feasible to achieve finer grouping by incorporating additional criteria previously outlined, such as date, time, or geographic location. This approach to nuanced categorization is equally applicable to the other established groupings, allowing for more detailed and specific grouping of the images. Details of the FAHRIS grouping validation are reported in the “Method Validation” section.

### Stitching images

**Fig. 4:**
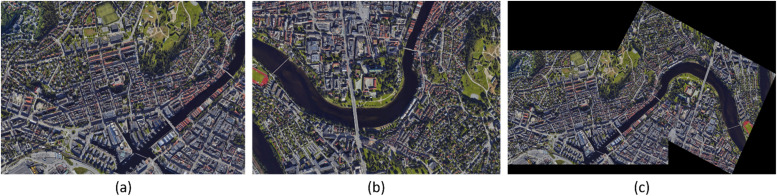
Illustration of stitched images of Trondheim city center (Norway) taken from Google Earth. (c) is the result of (a) and (b) through FAHRIS.

To create an overview of an area as it is monitored (e.g. by cameras equipped to a radiosonde or UAV), acquired images can be stitched together into one image. Through feature-based image alignment, key features of two images can be compared, allowing for image adjustments and matching. The key features are found through OpenCV's Oriented FAST and Rotated BRIEF (ORB), an algorithm for feature detection and description with rotation invariance. The rotation invariance is achieved by orienting keypoint-patches based on intensity weighted centroids, guiding the sampling pattern, and ensuring consistent feature identification regardless of the image rotation.^6^ They are then brute-force matched before utilizing a RANSAC algorithm to evaluate a homograph matrix, and then using this matrix to calculate the image transformation.^7^ An example of this stitching technique is illustrated in Fig. 4. In FAHRIS, this ORB algorithm is implemented and utilized during processing. During operation, FAHRIS takes each previously classified group and stitches them together into a final image, exemplified in Fig. 4(c) using the ORB algorithm.

**Fig. 5:**
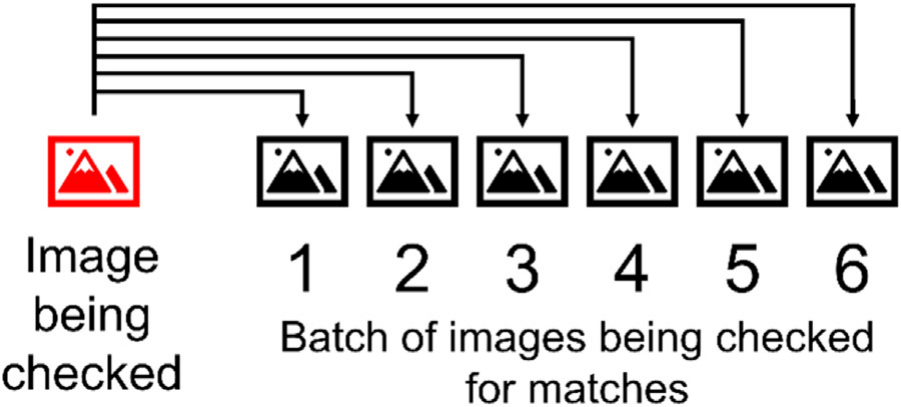
Illustration of the identification of matches within a batch.

The FAHRIS stitching algorithm processes the data organized in grouped folders. This employs a stitching procedure for image integration, as aforementioned. Initially, it selects the first file within the grouped folder to undergo stitching, inspecting datasets of six images sequentially for potential matches, illustrated in Fig. 5. Upon identifying one or more matches within a dataset, the algorithm stitches these images, subsequently relocating them to a specially designated folder named “result_x” along with the stitched result image. Images without matches remain in the original grouped folder for subsequent analysis.

If the initial dataset yields no matches, the algorithm proceeds to the subsequent dataset of six images and continues this process until a match is found or all images in the folder have been examined. After the complete evaluation, unmatched images are transferred to a folder for failed attempts. The procedure then applies to the next file in line within the original grouped folder, continuing iteratively through the entire grouped folder. Details of the FAHRIS stitching validation are reported in the “Method Validation” section.

## Method validation

### Classification

**Fig. 6:**
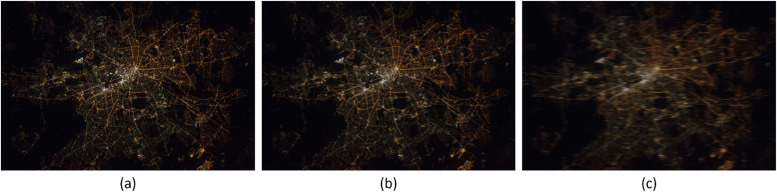
Illustration of generated test images to validate the classification capability of FAHRIS from an image of Berlin at night.^8^ (a) Source image (no blur). (b) Slightly blurred image classified as accepted by FAHRIS. (c) Blurred image classified as rejected by FAHRIS.

The FAHRIS classification system's capability to handle motion blur was validated through a series of tests that involved applying varying levels of generated motion blur to a source image. This was done through making an algorithm designed to generate a test set through the application of different levels of motion blur to a source picture. This provided insights into the impact of different motion blur severities. As a result, a threshold value of 160 was established for classifying images in FAHRIS. Fig. 6 illustrates this validation. The source image is shown in (a), a slightly blurred image classified as “accepted” by FAHRIS is shown in (b), and a blurred image classified as “rejected” by FAHRIS is shown in (c).

### FAHRIS validation analysis

The analysis was conducted on a Lenovo Yoga Slim 7i Pro 14 laptop connected to a power -source during processing. The technical specifications of the laptop are listed below:

• Processor: 11th Generation Intel® Core™ i7–11370H
• Memory: 16 GB LPDDR4X-4266MHz
• Graphics: NVIDIA® GeForce® MX450 2GB GDDR6
• Storage: 512 GB SSD M.2 2280 PCIe TLC
• OS: Windows 11 Home 64

The analysis used data acquiring during three flight sessions in respective monitoring campaigns carried out in Central and North-Eastern Italy in the cities of “Florence”, “Treviso”, and “Padua” respectively. Information about these flights can be found below in Table 2.

**Table 2:** Information about the data acquisition flights.

Data set	Flight type	Location	Date of flight	Instrumentation
Florence	Balloon	Central Italy, Florence, Certaldo	06.07.2021	Sounding balloon [2]
Treviso	Drone	North-Eastern Italy, Treviso, Pieve di Soligo	28.11.2023	DJI Mavic 2 [7]
Padua	Drone	North-Eastern Italy, Padua, Ponte San Nicolò	26.10.2022	DJI Mavic 2 [7]

The image files from the flights were first processed through the FAHRIS classification system to assess motion blur beyond the predetermined threshold. Following this initial classification, the accepted images were organized into four groups:

• Group 1: Date
• Group 2: Time
• Group 3: Geographical location
• Group 4: Altitude

The outcomes from the analysis of these flights are summarized in Table 3 (Florence), Table 4 (Treviso), and Table 5 (Padua) respectively. These tables detail the FAHRIS classification results, and the criteria used for grouping the images.

**Table 3:** Results from the classification of the Florence dataset. From 1314 images in the dataset, 366 were classified as acceptable. The remaining of the classified images consist of 88.2 % false positives. Due to missing EXIF-data in this dataset, neither grouping nor stitching was performed for this dataset.

Florence		
Acquisition flight type: Balloon	No. Images: 1314	Acceptable Classified images: 366

**Table 4:** Results from the classification of the Trevisodataset acquired by an unmanned aerial vehicle (i.e. a drone). From 132 images in the dataset, 101 were classified as acceptable.

Treviso							
Group Type	Range (Type, From, To)			Group Result	Stitched Images (Used)		Stitch duration
Group 1	Date	28.11.23	28.11.23	101	13	(31)	33 min 38s
Group 2	Time	18:30	18:44	41	12	(29)	5 min 42s
	Time	18:45	18:59	24	0	(0)	2 min 48s
	Time	19:00	19:14	36	1	(2)	5 min 50s
Group 3	LAT, LON	00–20	00–20	N/A	N/A	N/A	N/A
	LAT, LON	00–20	20–40	47	9	(23)	7 min 30s
	LAT, LON	20–40	00–20	N/A	N/A	N/A	N/A
	LAT, LON	20–40	20–40	54	4	(8)	11 min 36s
Group 4	Height	150	199	44	0	(0)	8 min 55s
	Height	200	249	49	11	(23)	7 min 12s
	Height	250	300	8	2	(8)	2 min 13s
Acquisition flight type: Drone				No. Images: 132		Classified images: 101

**Table 5:** Results from the classification of the Padua dataset acquired by an unmanned aerial vehicle (i.e. a drone). From 46 images in the dataset, 45 were classified as acceptable.

Padua							
Group Type	Range (Type, From, To)			Group Result	Stitched Images (Used)		Stitch duration
Group 1	Date	26.10.23	26.10.23	45	10	(20)	4 min 9s
Group 2	Time	20:00	20:04	10	0	(0)	0 min 24s
	Time	20:05	20:09	14	5	(10)	0 min 52s
	Time	20:10	20:14	21	5	(10)	1 min 7s
Group 3	LAT, LON	45–49	40–44	45	10	(20)	8 min 36s
Group 4	Height	0	49	5	0	(0)	0 min 6s
	Height	50	99	5	0	(0)	0 min 6s
	Height	100	149	6	2	(4)	0 min 59s
	Height	150	199	4	0	(0)	0 min 5s
	Height	200	249	10	2	(4)	1 min 38s
	Height	250	299	6	1	(2)	0 min 27s
	Height	300	249	9	0	(0)	0 min 50s
Acquisition flight type: Drone				No. Images: 46		Classified images: 45	

## Limitations

A method to detect heading was implemented into FAHRIS by making a function that analyses geographical movements, as the EXIF metadata did not provide the heading information of the image. This function arranges the images in chronological order based on capture time and uses the sequence of images to estimate the direction of capture by observing changes in longitude and latitude. However, this method does not determine the heading of the first image due to the absence of a positional delta. The accuracy of the headings obtained through this approach has not been validated as actual heading data was not recorded during the flights.

As discussed in the stitching images paragraph in the FAHRIS Method details section, a procedure for processing datasets was introduced, restricted to handling only six images at a time. This limitation was implemented to avoid errors due to the constraints of the available computational resources, particularly to prevent exceeding the computer's memory capacity when processing more than six images simultaneously. While this approach effectively reduced the occurrence of these errors, the procedure of processing datasets increased processing time compared to data folders that previously could be processed without processing datasets with six images at a time.

During the processing of the image files captured in the test using a balloon (Table 3), challenges were encountered with the classification algorithm. Typically, files are processed with a blur threshold set at 160, however, this setting proved to be inadequate for these image files. The initial application of this threshold resulted in the classification of only five images, all of which were blurred and captured from the ground at the moment of deployment. This issue necessitated a revised approach to accurately classify the intended aerial images.

To improve the classification accuracy within FAHRIS, the blur threshold was progressively lowered, improving the identification of stable images. However, this adjustment increased the risk of incorrectly classifying severely blurred images as acceptable. The variability in blur values among images further complicated the classification process: some stable images exhibited blur values lower than those typically observed in unstable images, and vice versa. After several iterations, a new threshold of 27.5 was determined to yield the highest percentage of correctly classified images. This optimal threshold was established through a manual review process, which validated its effectiveness in distinguishing between stable and unstable images.

To evaluate the performance of FAHRIS, the confusion matrix (Table 6) [14] was adopted. This matrix visualizes the accuracy of classifications across two key categories: True Positives (TP), where images are correctly identified as stable, and True Negatives (TN), where images are correctly identified as blurred. Similarly, the misclassifications are also detailed, with False Positives (FP) representing stable images incorrectly classified as blurred, and False Negatives (FN) representing blurred images incorrectly classified as stable.

**Table 6:** Confusion matrix for binary classification.

		Actual	
		Positive	Negative
Predicted	Positive	True Positive (TP)	False Positive (FP)
	Negative	False Negative (FN)	True Negative (TN)

Subsequently, the performance metrics of accuracy, precision, recall, and F1 score have been calculated to assess the classifier's effectiveness. **Accuracy** (Eq. (1)) measures the proportion of correctly identified cases out of the total cases examined, reflecting the classifier's overall effectiveness. **Precision** (Eq. (2)) indicates the reliability of positive predictions by measuring the ratio of true positives to all the positive classifications made by the classifier. **Recall** (Eq. (3)), also known as sensitivity, reflects the ability to detect all relevant instances, expressed as the ratio of true positives to the actual positives. The **F1 Score** (Eq. (4)) combines precision and recall into a single metric, providing a balance between them, where a score of 1 indicates perfect precision and recall. The equations [12] provided for each of these categories are listed below:

(1) $$Accuracy=\frac{TP+TN}{TP+TN+FP+FN}$$

(2) $$Precision=\frac{TP}{TP+FP}$$

(3) $$Recall=\frac{TP}{TP+FN}$$

(4) $$F1\;score=2\cdot \frac{Precision\cdot Recall}{Precision+Recall}$$

**Fig. 7:**
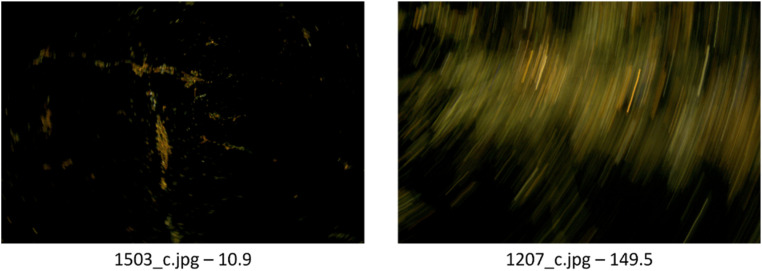
Comparison between two falsely classified images from the Florence dataset. 1503_c is a false negative. 1207_c is a false positive. In each image, the file name is to the left, while the blur value is to the right. The 1503_c.jpg has a 14 times increased blur value compared to the 1207_c, while having little blur. Meanwhile, the 1207_c has a blur value that should indicate little blur – while extremely blurred.

Table 7 presents the results from the FAHRIS confusion matrix and the performance metrics for the original and adjusted threshold values applied to the “Florence” files in addition to the “Treviso” and “Padua” acquired files. Notably, among the adjusted “Florence” files, there were five false negatives with blur values of 1.5, 13.1, 10.9, 26.8, and 26.9, respectively. Fig. 7 illustrates two significant cases: to the left, “1603_c.jpg”, an image excluded as acceptable with a blur value of 10.9, and to the right, “1207_c.jpg”, an extremely blurred image wrongly classified as acceptable with a blur value of 149.5. A further complication in processing the “Florence” files was the absence of EXIF metadata, preventing the organization of images based on metadata attributes (i.e. grouping).

**Table 7:** Results of FAHRIS confusion matrix and performance metrics of the images files (at both the original threshold of 160 and the adjusted optimal one at 27.5), and the Trevisoand Padua data sets at a threshold value of 160. T_val_ = threshold value used for the classification.

Data set	T_val_	TP	TN	FP	FN	Accuracy	Precision	Recall	F1 Score
Florence (Orig.)	160	0	1268	41	5	96.5 %	0.00 %	0.00 %	0.00 %
Florence (Adj.)	27.5	41	943	5	325	74.9 %	89.1 %	11.2 %	19.9 %
Treviso	160	101	0	31	0	76.5 %	76.5 %	100 %	86.7 %
Padua	160	45	1	0	1	95.7 %	100 %	97.8 %	98.9 %

**Fig. 8:**
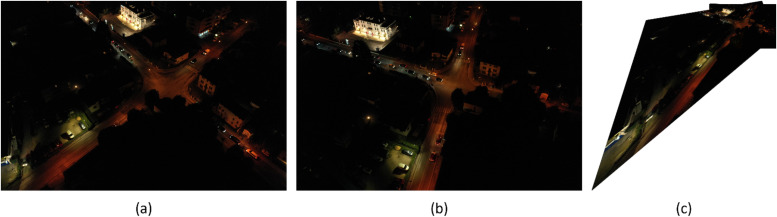
Result 11 from the Trevisodataset, FAHRIS grouping by altitudes between 200 and 249m. (a) and (b) have been stitched together in (c).

**Fig. 9:**
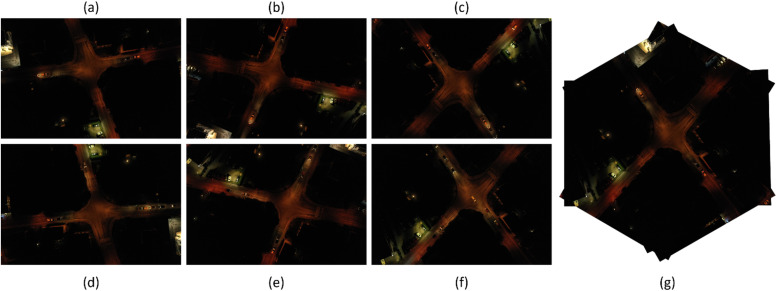
Result 1 from the Trevisodataset, FAHRIS grouping by altitudes between 250 and 300m. (a) to (f) have been stitched together in (g).

**Fig. 10:**
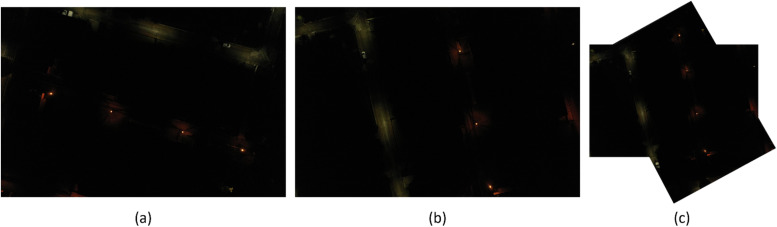
Result 2 from the Trevisodataset, FAHRIS grouping by altitudes between 250 and 300m. (a) and (b) have been stitched together in (c).

**Fig. 11:**
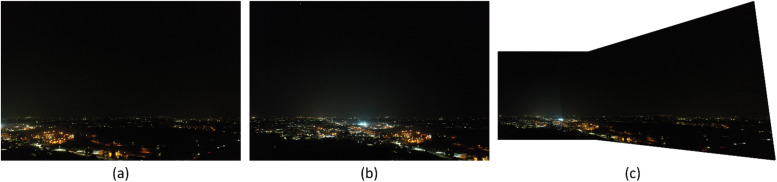
Result 1 from the Padua dataset, FAHRIS grouping by altitudes between 100 and 149m. (a) and (b) have been stitched together in (c).

**Fig. 12:**
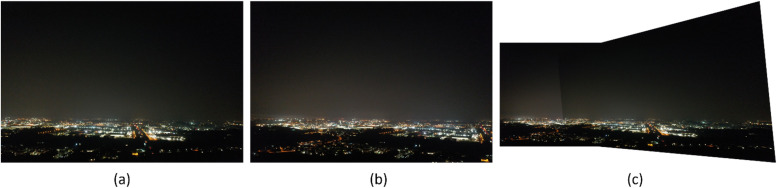
Result 2 from the Padua dataset, FAHRIS grouping by altitudes between 100 and 149m. (a) and (b) have been stitched together in (c).

**Fig. 13:**
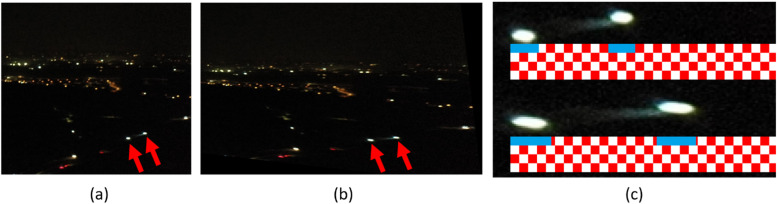
Comparison of the bottom-right corner of the two images in Fig. 12. The red arrows on the figure point out two points that have been stretched in the stitched image (b). A local estimation into the stretching of these two light sources was conducted by taking the pixel width of each light source and finding the percentage difference. This can be seen in (c), where the top image comes from (a), and the bottom one from (b).

Results from the image stitching process in FAHRIS are illustrated in Fig. 8, Fig. 9, Fig. 10, Fig. 11, Fig. 12. A notable limitation, as demonstrated in Fig. 8, Fig. 11, is the stretching of certain images where there is a significant relative angle between the stitched images. Despite improvements in Fig. 11, Fig. 12 compared to Fig. 8, a limitation of image warping remains apparent, with visibly stretched sections, particularly evident at the extreme right of the stitched images (as shown in part (c) of Fig. 11, Fig. 12). This issue is highlighted further in Fig. 13 which provides a detailed comparison, showcasing the bottom-right corner of the stitched images in Fig. 12 due to the orientation and angle of the original images.

In part (c) of Fig. 13, a grid ruler was applied where each square (white or red) represents a 5 × 5 pixel area. The top image is from (a) and the bottom part from (b) in Fig. 13, indicating stretching percentages of 144 %, 147 % and 151 % for the bottom left, bottom right and between the two sources, respectively. The red arrows in (a) and (b) highlight specific points that have undergone stretching in the stitched image. Such distortions can lead to inaccuracies in subsequent image processing steps, such as changes in the perceived colors intensity or light distribution due to the enlarged pixel areas.

## Declaration of competing interest

The authors declare that they have no known competing financial interests or personal relationships that could have appeared to influence the work reported in this paper.

## Data Availability

Data will be made available on request.

## Appendix Supplementary materials

Supplementary material *and/or* additional information [OPTIONAL] Image, application 1

## Footnotes

8 https://urbannext.net/satellite-images-better-cities/.

## Footnotes

1 https://www.dji.com/no/mavic-3-pro.

2 https://www.dji.com/no/mavic-2/info.

3 https://docs.opencv.org/3.4/d1/dfb/intro.html.

4 https://docs.opencv.org/3.4/d5/db5/tutorial_laplace_operator.html.

5 https://pyimagesearch.com/2015/09/07/blur-detection-with-opencv/.

6 https://docs.opencv.org/4.x/d1/d89/tutorial_py_orb.html.

7 https://datahacker.rs/005-how-to-create-a-panorama-image-using-opencv-with-python/.
